# Development of an Ultrasound-Assisted Extraction Procedure for the Simultaneous Determination of Anthocyanins and Phenolic Acids in Black Beans

**DOI:** 10.3390/foods12193566

**Published:** 2023-09-26

**Authors:** Valentina Melini, Francesca Melini, Francesca Luziatelli, Maurizio Ruzzi

**Affiliations:** 1 CREA Research Centre for Food and Nutrition, Via Ardeatina 546, I-00178 Roma, Italy; francesca.melini@crea.gov.it; 2 Department for Innovation in Biological, Agrofood and Forest systems (DIBAF), University of Tuscia, Via C. de Lellis, snc, I-01100 Viterbo, Italy; f.luziatelli@unitus.it (F.L.); ruzzi@unitus.it (M.R.)

**Keywords:** black beans, legumes, phytochemicals, phenolic compounds, anthocyanins, response surface methodology, ultrasound-assisted extraction, GRAS solvent

## Abstract

Beans are an essential source of nutritional components such as plant proteins, minerals and dietary fiber, as well as of antioxidants such as phenolic compounds. Phenolic compounds are praised for their biological activities and possible benefits on human health. Since no official methods are available for phenolic compound extraction, the optimization of extraction parameters via Response Surface Methodology (RSM) has become a commonly used methodological approach for reliable determinations. This study aimed to apply RSM to optimize the ultrasound-assisted extraction procedure of phenolic compounds, including anthocyanins, from black beans. A Generally Recognized As Safe solvent (ethanol) was used. Solvent concentration, extraction time, and solvent/sample ratio were optimized to maximize two responses: Total Anthocyanin Content (TAC) and Total Phenolic Content (TPC). An ethanol concentration of 64%, 30 min extraction time, and a 50 mL/g solvent/sample ratio were identified as the optimal extraction conditions. The TAC was 71.45 ± 1.96 mg cyanidin-3-O-glucoside equivalents 100 g^−1^ dm, and the TPC was 60.14 ± 0.89 mg gallic acid equivalents 100 g^−1^ dm. Among the pigmented phenolic compounds, cyanidin-3-O-glucoside and peonidin-3-O-glucoside were identified in the extracts. Regarding phenolic acids, caffeic, sinapic, and t-ferulic acids were detected.

## 1. Introduction

Legume production and consumption can make an important contribution to achieving some of the goals set by the 2030 Agenda for Sustainable Development [1]. Legumes have low greenhouse gas and water footprints compared to meat production; they enrich soil through nitrogen fixation, and they present a notable degree of biodiversity, with high levels of variation with respect to growth habitats, seed characteristics (i.e., size, shape, color), maturity, and adaptation. 

Common bean (*Phaseolus vulgaris*) is one of the most important legumes [2] with remarkable variability (>40,000 varieties) [3]. In a sustainable development framework, growing local ecotypes allows for the diversification of agri-food systems and facilitates sustainable diets. Common bean is a rich source of plant proteins, complex carbohydrates, dietary fiber, and minerals; it is low in fat [4] and cholesterol- and gluten-free. It also contains biologically active phytochemicals [5]. 

Phenolic compounds (PCs) are phytochemicals found in most plant tissues. They are secondary plant metabolites that are specifically synthesized via the shikimic acid and phenylpropanoid pathways [6]. PCs are not nutrients, but they possess several bioactive properties, and increasing science-based evidence has shown that their dietary intake provides health-protective effects due to their antioxidant, anti-inflammatory, hypoglycemic, hypolipidemic, and anticarcinogenic activity [5]. Studies have also increasingly shown that PCs play a key role against the onset of neurodegenerative diseases [7] and in the prevention of cardiovascular diseases (CVDs) and some types of cancers [8]. 

Among phenolic compounds, anthocyanin pigments have also attracted interest for their positive function(s) in human health. Being responsible for the pigmentation of many plant foods, including fruit and vegetables, cereals, legumes, and pseudocereals [9,10,11], anthocyanins have beneficial effects in CVDs and neurodegenerative disorders and also possess anticarcinogenic activity [12]. They are also valuable for weight management and polygenic disorders like hypertension, coronary heart disease, and diabetes [12].

In plant foods, phenolic compound content and composition are highly diverse, reflecting the diversity of plant composition as well as the reaction of plant phenolics during storage and processing. Among them, pigmented beans are particularly rich in polyphenols, flavonoids, and anthocyanins, which also contribute to seed coat color and sensory characteristics [9,11,13,14,15].

At a time when the discussion on the role of phytochemicals in human health is becoming more and more important, phenolic compounds must be investigated in food matrices. However, there is no standard method for the quantitative and qualitative determination of phenolic compounds in foods; the choice of methodology is very complex. In particular, the first step for reliable quantitation of phenolic compounds by colorimetric assays and profile determination by chromatographic techniques is related to the identification of the most effective sample preparation and extraction procedure. This ensures that an accurate determination of polyphenols is possible. 

The prolific literature that have appeared in the literature over the last years have shown that a considerable number of methods are currently available to determine phenolic compounds in food, but the development of extraction methods is often a one-parameter-at-a-time proposition. This approach is time-consuming and does not consider the interactions among variables. In an attempt to overcome the limitations of single-parameter optimization and identify an approach that allows for the optimization of all of the factors involved in the extraction step (e.g., temperature, solvent/sample ratio, solvent concentration, time, etc.) based on their interactions, Response Surface Methodology (RSM) has emerged as an effective statistical tool [16,17,18,19,20,21]. Moreover, several extraction procedures are based on conventional approaches such as shaking. On the contrary, the application of techniques, such as ultrasound-assisted extraction (UAE), microwave-assisted extraction, and supercritical fluid extraction enables one to reduce extraction time, decrease solvent consumption, and use environmentally friendly solvents, resulting in lower levels of energy consumption [22].

Amidst the backdrop of sustainable food consumption and the emergence of short food supply chains, consumers have shown a growing interest in local foods. Local foods are preferred to imported products for their “locality”, “seasonality”, and “sensorial quality”. However, little is known about their nutritional properties and their contribution to the intake of bioactive compounds. This study aimed to develop an UAE procedure to extract phenolic compounds in black beans via RSM and the use of green solvents to determine anthocyanins and phenolic acids in black beans with local relevance.

## 2. Materials and Methods

### 2.1. Chemicals

The following chemicals were purchased from Carlo Erba Reagents (Milan, Italy): acetic acid, acetonitrile, citric acid, ethanol, Folin–Ciocalteu’s Reagent (FCR), *n*-hexane, potassium chloride, sodium acetate, sodium carbonate, and trichloroacetic acid. 

Phenolic compound standards (i.e., gallic acid, protocatechuic acid, catechin, p-hydroxybenzoic acid, chlorogenic acid, vanillic acid, epicatechin, caffeic acid, syringic acid, p-coumaric acid, t-ferulic acid, sinapic acid, rutin, cyanidin-3-O-glucoside, peonidini-3-O-glucoside, and malvidin) were purchased from Sigma-Aldrich (Milan, Italy) and Extrasynthèse (Geney, France). The aqueous solutions used in HPLC analysis were prepared with water that had been purified using a Milli-Q system (Millipore Corp., Billerica, MA, USA). All reagents and solvents were HPLC- or analytical-grade.

### 2.2. Sampling

Black beans (*Phaseolus vulgaris* L.) were sampled from plants grown in the Tuscia geographical area (Viterbo, central Italy) and made available just upon harvest in September 2021. The black bean samples were packaged under a protective atmosphere, and no seeds were broken or damaged. Upon arrival at the CREA Research Centre for Food and Nutrition laboratory, the test samples were immediately prepared for analysis. Specifically, the seeds were ground using an analytical batch mill (Janke and Kunkel IKA Labortechnik, Staufen, Germany), and an ASTM woven wire mesh sieve No. 18 was used to ensure the uniform granulometry of the obtained powder. 

All ground samples were defatted immediately before analysis via blending with *n*-hexane (1:5 *w*/*v*, 5 min, three-fold vortex) at room temperature [23].

### 2.3. Response Surface Methodology

To obtain a model for optimizing the UAE of anthocyanins and phenolic acids in black beans, an experimental design was created using the Design Of Experiments (DOE) tool of the Minitab Pro 18 software.

#### 2.3.1. Design of Experiments: Variable Selection

In this study, a three-level/three-factor Box–Behnken Design (BBD) was used. Three factors, namely, solvent concentration (X_1_), extraction time (X_2_), and solvent/sample ratio (X_3_), were selected as independent variables. All variables were fixed at 3 levels, namely, low level (−1), midpoint (0), and high level (+1), with X_1_ being 50, 65, and 80%; X_2_ being 10, 20, and 30 min; and X_3_ being 20, 35, and 50 mL g^−1^ (Table 1). The values were established based on earlier experiments and investigations [9,10]. The extraction temperature was kept constant at 40 °C to protect the phenolic compounds from degradation.

Total Anthocyanin Content (TAC) and Total Phenolic Content (TPC) were set as responses.

#### 2.3.2. Box–Behnken Design and Regression Equation

A total of seventeen experiments with five center value replications and a randomized order were obtained (Table 1). Extraction runs were carried out according to the order established by the experimental design.

The following second-order polynomial equation was used to fit the experimental data of the studied variables:

$$Y=\beta_{0}+\sum_{i=1}^{k}\beta_{i}\;X_{i}+\sum_{i=1}^{k}\beta_{ii}\;X_{i}^{2}+\sum_{i=1}^{k}\;\sum_{j=1}^{k}\beta_{ij}\;X_{i}\;X_{j}\;$$

*Y* is the dependent variable (i.e., TAC and TPC); *X*_i_ and *X*_j_ are the independent variables (i.e., solvent concentration, extraction time, and SSR); *k* is the number of tested variables (*k* = 3). *β_n_* are the regression coefficients; *β*_0_ is for the intercept, *β*_i_ is for linear terms, *β*_ii_ is for quadratic terms, and *β*_ij_ is for interaction terms. 

The statistical significance of the terms in the regression equations was verified by using analysis of variance (ANOVA) for each response. Statistically non-significant (*p* > 0.05) terms were excluded from the mathematical model, and the experimental data were then fitted only to the significant (*p* < 0.05) parameters. The coefficient of determination R^2^ was also considered to estimate the quality of the fit of the polynomial model equation to the responses: an R^2^ value close to 1 in a model denotes that it has excellent prediction efficiency. The adjusted R^2^ (Adj. R^2^) was also estimated to test the model’s accuracy.

In the regression model, both *F*-value and lack of fit (LOF) were expressed at a probability (*p*) of 0.05. 

The responses obtained from the regression models were represented graphically as contour and three-dimensional plots.

#### 2.3.3. Verification of the Model

The optimal extraction conditions were validated for maximum TAC and TPC. Model validation was performed by extracting phenolic compounds at the optimal conditions and as per the analytical procedure followed in the experimental runs. The experimental data were compared to the predicted values to validate the model.

### 2.4. Ultrasound-Assisted Extraction of Phenolic Compounds

A three-step ultrasound-assisted extraction method was used. At each extraction step, an established amount (Table 1) of the test sample was put in PYREX™ screw cap culture tubes, and 5 mL of extraction solvent (i.e., ethanol aqueous solution acidified with citric acid) was added. As reported in the literature, weak acid was used for the acidification of the extraction mixture [24,25]. The culture tubes were transferred to an ultrasound bath system (Elmasonic S 100 H, Elma Schmidbauer GmbH, Singen, Germany) that operated at 37 kHz. The test tubes were allowed to acclimate to the water bath temperature for 2 min. In the extraction phase, the water bath temperature was controlled. The extraction conditions were set according to the experimental design provided by the Minitab Pro 18 (Minitab Inc., State College, PA, USA) software.

After each UAE step, the solid–liquid solution test tube was cooled down at +4 °C for 10 min. Afterwards, it was centrifuged at 7000 rpm for 10 min to recover the supernatant, which was transferred into another PYREX™ tube for the final combination of the supernatants of the three extraction steps. The second and third steps of the UAE were carried out according to the procedure specified for the first step. TAC and TPC were determined in an aliquot of the pooled extract after adding trichloroacetic acid to a final concentration of 15%, to precipitate proteins [26]. 

### 2.5. Spectrophotometric and Chromatographic Determination of Phenolic Compounds

After UAE, the TAC and TPC of the food-grade phenolic extracts were determined spectrophotometrically, as specified below. The tests were performed in triplicate. 

The phenolic compounds in the food-grade extract obtained at optimum conditions were determined via High-Performance Liquid Chromatography (HPLC).

#### 2.5.1. Total Anthocyanin Content

Total anthocyanin content was detected using the pH differential method (AOAC Official Method 2005.02) [27]. Briefly, the extract (200 µL) was first diluted to a factor of 1:5 *v*/*v* with potassium chloride buffer 0.025 M (pH adjusted to 1.0 with HCl) and sodium acetate buffer 0.4 M (pH adjusted to 4.5 with NaOAc/HCl), and absorbance was read at wavelengths of 520 and 700 nm, respectively, after 20 min. TAC was determined by calculating the absorbance of the diluted sample (*A*) as follows:

A = (A_λvis-max_ − A_700_)_pH 1.0_ − (A_λvis-max_ − A_700_)_pH 4.5_

The monomeric anthocyanin pigment concentration of the original sample was calculated by using the following formula:

$$Monomeric\;Anthocyanin\;pigment=\frac{A\times MW\times DF\times 1000}{\varepsilon \times l}$$

where *A* is the absorbance of the diluted sample, *MW* is the molecular weight, *DF* is the dilution factor, 1000 is the conversion factor (from g to mg), *ε* is the molar extinction coefficient (26,900 L⋅mol^−1^⋅cm^−1^), and *l* is the pathlength in cm (a pathlength of 1 cm is assumed). 

Results were expressed as mg of cyanidin-3-O-glucoside equivalents per gram of sample on a dry matter basis (mg C3GE 100 g^−1^ dm).

#### 2.5.2. Total Phenolic Content

TPC was determined spectrophotometrically using the colorimetric assay of the FCR as described by Sompong et al. [28] and Melini and Melini [10]. Briefly, a known amount of extract was transferred into a set of test tubes, and 600 µL of water-diluted FCR was added into each test tube. Three minutes later, sodium carbonate (Na_2_CO_3_ 75 g/L) (960 µL) was added to adjust pH to 10–10.5. The tubes were put into a laboratory water bath at 50 °C for 10 minutes. Absorbance was registered at 760 nm against the reagent blank.

TPC was quantitated using a calibration curve of pure gallic acid (concentration range: 1.1–12.3 µg mL^-1^). The regression equation was y = 0.1577x − 0.1033, with a coefficient of determination (R^2^) of 0.9902. The results are reported as milligrams of Gallic Acid Equivalents (GAE) per 100 g of sample on a dry matter basis (mg GAE 100 g^-1^ dm).

#### 2.5.3. Phenolic Compound Determination

The phenolic compounds present in the black bean extracts obtained under optimal UAE conditions were determined using a Varian ProStar HPLC equipment (Varian Inc., 2700 Mitchell Drive, Walnut Creek, CA, USA) provided with a photodiode array detector and controlled by Galaxie Chromatography Data System software (version 1.9.302.952).

The extract was evaporated till dryness by using a rotary evaporator (Bϋchi Rotavapor R-200, Milano, Italy). The residue was redissolved in aqueous methanol (50:50 *v*/*v*) and filtered through a 0.45 μm filter before HPLC analysis.

Phenolic compounds were separated using a reverse-phase Inertsil^®^ ODS-3 column (250 × 4.6 mm i.d., 5 µm; CPS analitica, Milano, Italy). The following mobile phases were employed for elution: (i) water acidified with acetic acid (2.5%) (Solvent A) and (ii) acetonitrile (Solvent B). A 48 min gradient elution at 40 °C and 1.0 mL/min flow rate were applied. The initial mobile phase was 95% A and 5% B. The initial mobile phase was 95% A and 5% B. The percentage of mobile phase B increased in three steps: from 5% to 10% over the course of 3 min (0–3 min); from 10% to 30% over the course of 18 min (10–28 min) and from 30% to 40% over the course of 10 min (28–38 min). It was held constant for 5 min (38–43 min). It decreased back to 5% over the course of 5 min. Chromatograms were recorded at two wavelengths: 320 and 520 nm. Phenolic acids and anthocyanins were identified by comparing the sample’s retention time and UV-VIS spectra with pure reference standard that had been eluted under the same chromatographic conditions. 

### 2.6. Statistical Analysis

The experimental design was provided by Minitab Pro 18 (Minitab Inc., State College, PA, USA). The same software was also used to elaborate data (e.g., ANOVA, Response Surface Methodology). Contour plots and surface 3D graphs were obtained via the use of Design Expert software (version 10, Stat-Ease, Inc., Minneapolis, MN, USA). Experimental data were processed by using Microsoft® Excel® for Windows 365 (version 2103).

## 3. Results and Discussion

### 3.1. Fitting the Model with Experimental Data

The experimental data on TAC and TPC, which were obtained by using the different combinations in the 17 runs of the BBD, are shown in Table 2.

#### 3.1.1. Total Anthocyanin Content

Total Anthocyanin Content ranged from 25.41 ± 0.23 (Run 7) to 86.20 ± 0.63 mg C3GE 100 g^−1^ dm (Run 8). The lowest value was observed when the sample (SSR = 50 mL g^−1^) was extracted using acidified ethanol/water 80:20 (*v*/*v*) for 20 min (Table 1). The highest TAC was obtained upon extraction with 65% acidified aqueous ethanol, 30 min duration and a solvent-to-sample ratio of 20 mL g^−1^.

The experimental data obtained for TAC in the food-grade extracts were fitted to the second-order polynomial model equation, and ANOVA was used to determine the significance of the coefficients. Table 3 shows the regression coefficients and the *p* values which indicate the statistical significance of the association between the term and the response.

According to the ANOVA, the model for TAC is significant (*p* < 0.05), and there is only a 0.05% chance that the F-value (17.41) occurred because of noise. The ANOVA results also showed the significant (*p* < 0.05) linear effect of solvent concentration (X_1_) and extraction time (X_2_) on TAC, the significant (*p* < 0.001) quadratic effect of the two variables ($X_{1}^{2}$ and $X_{2}^{2}$), and the significant (*p* < 0.05) interactive effect of X_1_ X_2_ and X_2_ X_3_ on TAC. Based on the linear and quadratic regression coefficients for extraction time and the two-way interaction regression coefficient for solvent concentration and time (β_12_), these variables significantly increased TAC. The linear regression coefficients for solvent concentration (β_1_) and solvent/sample ratio (β_3_), the quadratic regression coefficient for solvent concentration (β_1_^2^), and the two-way interaction regression coefficients for X_1_, X_2_, and X_3_ (β_12_ and β_23_) showed a negative effect (Table 3). ANOVA for the lack of fit test was not significant (*p* > 0.05). This indicates that the model fitted the data (Table 3). The R^2^ value revealed a high correlation between the response and the independent variables. The model explained 95.72% of data variance (Adj. R^2^ = 0.90; Table 3).

Taking into account only the significant factors, the following equation expresses the model showing the relationship between TAC and the extraction variables used:

(1) $$Y=54.94-4.83\;X_{1}+8.27\;X_{2}-18.43\;X_{1}^{2}+17.39\;X_{2}^{2\;}+6.05\;X_{12}-7.21\;X_{23}$$

The statistical significance of the regression equation for TAC was checked using Fisher’s F-test. The F-value of the regression coefficients was compared to the tabulated one to verify if the model variables have a significant (*p* < 0.05) effect on TAC response, and it was observed that F_regression_ (17.41) was higher than the tabulated value (F_tabulated(9,5,0.05)_ = 3.48) and that the *p*-value was lower than 0.05. This indicates that the model variables significantly affect TAC response at a 95% confidence level. Regarding the F-value of the LOF, it was compared to the tabulated F-value, and it emerged that F_lack-of-fit_ (0.55) was lower than the tabulated value (F_tabulated__(3,2,0.05)_ = 19.16), meaning that the LOF statistics were not significant (*p* > 0.05); therefore, the model is valid.

**Figure 1:**
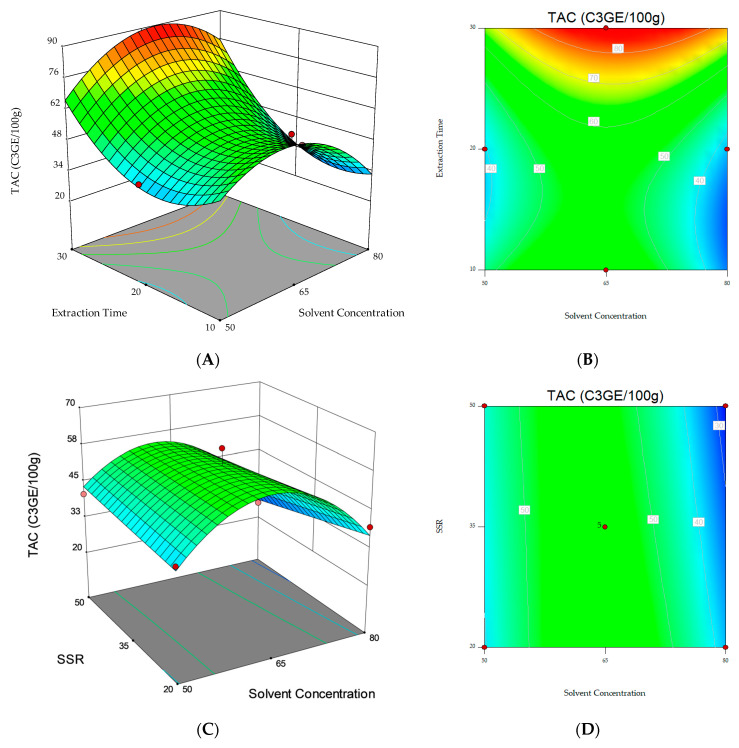

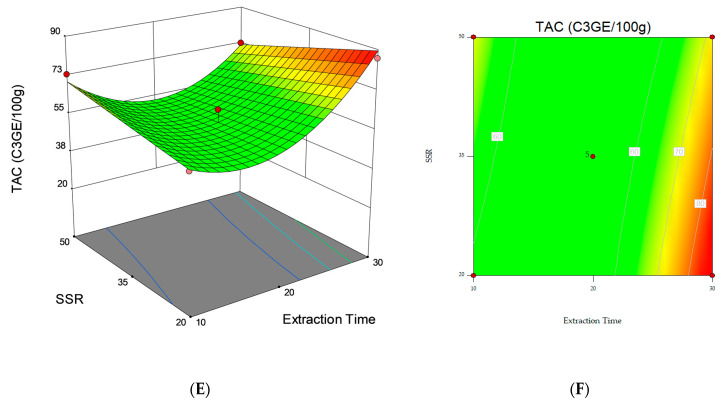
3D response surfaces and contour plots of Total Anthocyanin Content (TAC) as a function of the interaction between the dependent variables: (**A**,**B**) Solvent concentration and extraction time. (**C**,**D**) Solvent concentration and solvent/sample ratio (SSR). (**E**,**F**) Extraction time and solvent/sample ratio (SSR).

Response surface and contour plots were also elaborated to visualize both the optimum level that each variable should have for maximum TAC response and the combined effect of the different variables on the content of total anthocyanins (Figure 1). The graphical representation allowed for the visualization of the relationship between the experimental levels of the independent variables and the response. The non-plotted variable is kept at the midpoint level (0). Figure 1A,C, where significant (*p* < 0.05) variables interactions are represented, show that the highest TAC was obtained when the extraction was performed with an ethanol/water ratio (*v*/*v*) of 65:35 for 30 min (Figure 1A) and when a solvent/sample ratio of 20 mL g^−1^ and 30 min extraction time (Figure 1C) were applied. However, the results obtained in this study point out that it is possible that longer extraction times should be investigated to further maximize the response under analysis.

#### 3.1.2. Total Phenolic Content

Total Phenolic Content ranged between 31.95 ± 1.35 (Run 1) and 57.87 ± 2.19 mg GAE 100 g^−1^ dm (Run 15). The highest value was obtained when extraction was performed for 30 min with an ethanol/water ratio of 65:35 (*v*/*v*) and a SSR of 50 mL g^−1^. The lowest TPC value was found when raw black beans were extracted for 20 min with 50:50 (*v*/*v*) ethanol/water ratio and a SSR of 20 mL g^−1^ (Table 2).

The data obtained in the 17 runs of the BBD set in this study were fitted to the model equation specified above, and ANOVA was also performed for TPC. Table 3 shows that, with an F-value of 6.67, the model for TPC is also significant (*p* < 0.05). 

Regarding the model terms, the linear regression coefficients of extraction time (X_2_) and solvent/sample ratio (X_3_), the quadratic regression coefficients for solvent concentration ($X_{1}^{2}$) and extraction time ($X_{2}^{2}$), and the two-way interaction regression coefficient for Extraction Time × Solvent/Sample ratio (X_23_) show that these terms are significant (*p* < 0.05) (Table 3). Except for the quadratic coefficient for solvent concentration, the aforesaid regression coefficients show a significant positive effect on TPC (Table 3).

With an F-value of 0.20, ANOVA for the LOF test was not significant (*p* > 0.05), indicating that the model adequately fitted the data obtained in the experiments. The multiple determination coefficients (R^2^) revealed a good correlation between the response and the independent variables. The model explained 89.56% of the variance in the data (Table 3).

Based on only the significant terms, the following equation was obtained to express the relationship between TPC and the extraction parameters:

(2) $$Y=43.01+2.47\;X_{2}+4.46\;X_{3}-6.81\;X_{1}^{2}+3.37\;X_{1}^{2}+3.46\;X_{2}X_{3}$$

Fisher’s F-test was used to check the statistical significance of the regression equation for TPC. As shown in Table 3, the F-value of regression coefficients (F_regression_ = 6.67) is higher than the tabulated value F_tabulated(9,5,0.05)_ = 3.4817) (*p* < 0.05). This implies that the variables of the model have a significant effect on TPC response at 95% confidence level. In addition, the ratio of the mean square of LOF and pure error was lower than the tabulated value (F_lack-of-fit_ = 2.46 < F_tabulated__(3,2,0.05)_ = 19.16); this indicates that the LOF statistics were not significant (*p* > 0.05). Therefore, the model is valid.

**Figure 2:**
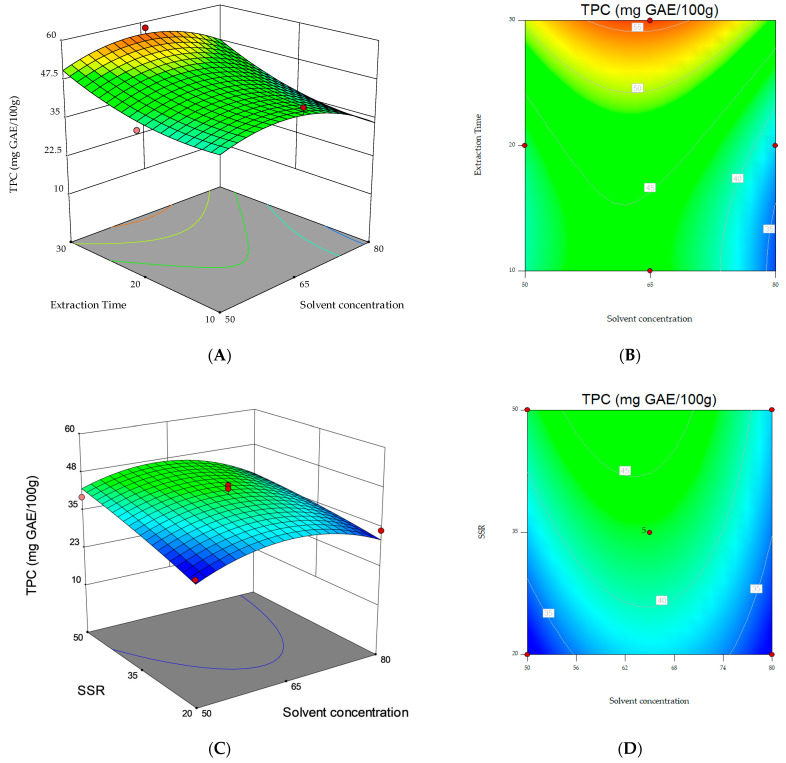

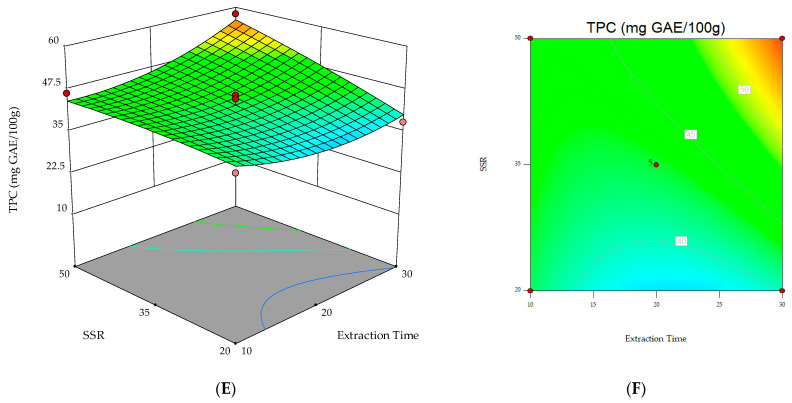
3D response surfaces and contour plots for TPC as a function of the interaction between the three variables. (**A**,**B**) Solvent concentration and extraction time; (**C**,**D**) solvent concentration and solvent/sample ratio (SSR); (**E**,**F**) extraction time and solvent/sample ratio (SSR).

Regarding the 2D contour and 3D surface plots, Figure 2 shows the response surfaces for TPC as a function of the interaction between the variables. The non-plotted variable is kept at its midpoint level (0). The 3D plot in Figure 2C, showing the two significant model terms of extraction time (X_2_) and SSR (X_3_), shows that the highest values for the response variable (i.e., TPC) were obtained when 30 min extraction time and 50 mL g^−1^ SSR were applied. 

However, the results obtained in this study prompt further investigations. Based on the 3D plot in Figure 2C, it can be speculated that longer extraction times or higher SSRs should be investigated. 

#### 3.1.3. Model Validation

Following the regression analysis, the UAE conditions were optimized for the maximum responses. The optimum experimental conditions for the maximization of the two responses (i.e., TAC and TPC) were a solvent concentration of 64%, 30 min extraction time, and 50 mL g^−1^ SSR. The model estimates for the optimum values of TAC and TPC were 64.98 mg C3GE 100 g^−1^ dm and 55.41 mg GAE 100 g^−1^ dm, respectively.

To validate the model, the UAE of the black beans was performed at the predicted optimum conditions using a GRAS solvent and following the method described for the BBD runs. 

Regarding TAC, the value obtained under optimal conditions was 71.45 ± 1.96 mg C3GE 100 g^−1^ dm; this value falls within the 95% Confidence Interval (CI), that is, 61.97–81.96 mg GAE 100 g^−1^ dm. The TPC mean value was 60.14 ± 0.89 mg GAE 100 g^−1^ dm; this value falls within the 95% CI (i.e., 50.09–62.11 mg GAE 100 g^−1^ dm). Based on these results, it was concluded that the experimental values obtained for TAC and TPC under optimum conditions verify the models and confirm that the identified settings allow one to obtain the highest TAC and TPC in food-grade phenolic extracts of black bean.

The data obtained for TAC (71.45 ± 1.96 mg C3GE 100 g^−1^ dm) in our sample are in keeping with the values obtained for dry common black beans in the literature. Mojica et al. observed values ranging from 38 to 170 mg C3GE 100 g^−1^ dm in beans from Mexico [29], and Madrera et al. [30] reported a content of 44 mg C3GE 100 g^−1^ for monomeric anthocyanins in black coat beans. In contrast, the TAC obtained in this study at optimum conditions is higher than the content reported by Ombra et al. for two endemic ecotypes of black beans (i.e., Nero Acerra and Nero Frigento) from Southern Italy [31], where TAC ranged between 1.89 and 6.33 mg C3GE 100 g^−1^ dm.

The TPC obtained in this study (60.14 ± 0.89 mg GAE 100 g^−1^ dm) is, on the other hand, in keeping with the values reported by Ombra et al. [31], who found TPC values ranging between 60.13 and 60.42 mg GAE 100 g^-1^. On the other hand, a high level of variability regarding the TPC in common beans was reported in a review by Yang et al. [32], where TPC was 10- to 100-fold higher than the value observed in the sample under investigation in this study. Multiple factors, such as extraction time, temperature, solvent concentration, and SSR significantly affect phenolic compound yield [33]. Thus, it is possible that the differences in the extraction solvents and conditions might have also contributed to this variability. Ganesan and Xu found a value (1260 mg GAE 100g^−1^) for TPC that was higher than what is reported in this study [34].

### 3.2. Determination of Phenolic Compounds in Ethanolic Extracts via HPLC

**Figure 3:**
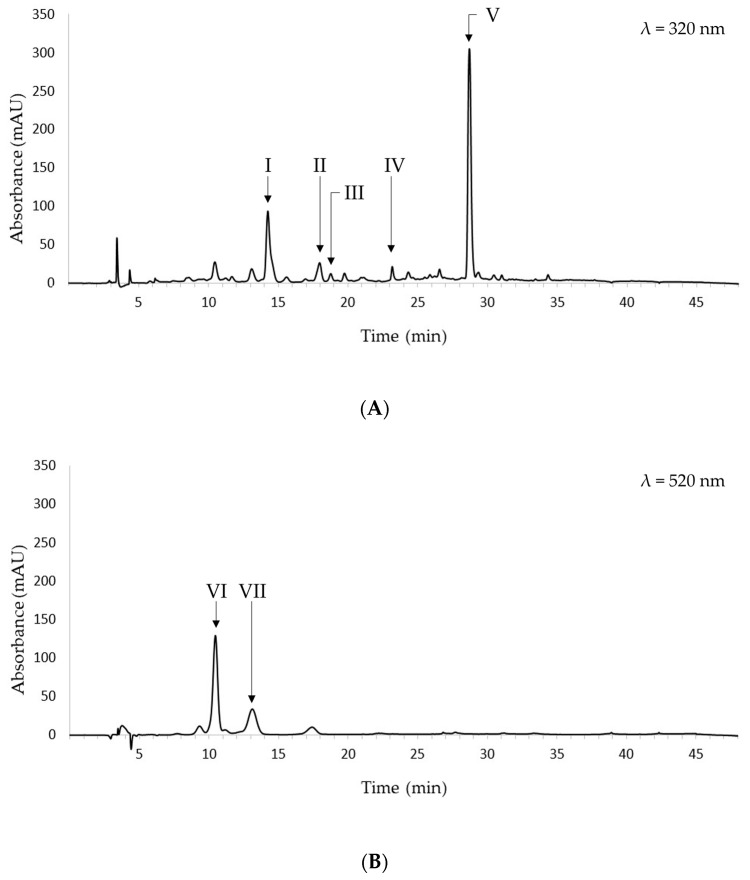
Chromatograms of the ethanolic extract obtained at optimal extraction conditions and recorded at 320 nm (**A**) and 520 nm (**B**). I: caffeic acid; II: t-ferulic acid; III: sinapic acid; IV: unknown; V: unknown; VI: cyanidin-3-O-glucoside; VII: peonidin-3-O-glucoside.

Figure 3 shows the representative chromatograms recorded at 320 nm and 520 nm of the black bean ethanolic extract obtained at optimal extraction conditions. 

The following phenolic acids were identified at 320 nm: caffeic, ferulic, and sinapic acids. The former was eluted at 14.27 min, ferulic acid was eluted at 18.00 min, and sinapic acid was eluted at 18.77 min. The relative areas were 74.4%, 19.6%, and 6.0%, respectively. Hence, caffeic acid was the most abundant among the identified hydroxycinnamic acids. Two additional peaks are present at 23.19 and 28.68 min. Based on their UV-VIS spectra and literature data, they may be tentatively identified as flavonols, namely, myricetin, quercetin, kaempferol, or glucosides thereof [35]. Comparisons between the phenolic acid content in the food-grade extract obtained in this study and that reported in current literature are usually limited by the fact that the selection of extraction solvent and conditions deeply affect the recovery of the phenolic compounds from the food matrix. Hence, inferences might be inaccurate and misleading. However, caffeic, ferulic, and sinapic acids have been found in extracts from beans investigated in recent studies. Caprioli et al. detected all of the above-mentioned phenolic acids in 70% ethanol extracts from beans [36]; Madrera et al. detected ferulic and sinapic acids in bean ethanolic extracts obtained via UAE [35], and Teixeira-Guedes et al. identified caffeic and ferulic acids in aqueous methanol extracts from bean samples [37]. 

It should be noted that the caffeic and ferulic acids were identified in both aqueous ethanol and methanol extracts. These data are in keeping with Melini and Melini [11], who observed no differences in the qualitative composition of phenolic compounds in ethanolic and methanolic extracts from quinoa.

The obtained food-grade extracts are thus promising from a nutritional point of view. The identified phenolic acids have several positive effects on human health. Caffeic acid has antiproliferative activity against cancer cells; it can alleviate diabetes, obesity, and metabolic syndrome. In addition, it has been found to counteract the symptoms of brain-related diseases, including Alzheimer’s disease and Parkinson’s disease [38]. Antioxidant, anti-inflammatory, anti-diabetic, cardioprotective, anti-cancer, and neuroprotective activities have also been reported for sinapic and ferulic acids [39,40].

As far as colored phenolic compounds are concerned, cyanidin-3-O-glucoside and peonidin-3-O-glucoside were identified in the ethanolic extracts under investigation. The former was eluted at 10.44 min, while the latter was eluted at 13.17 min. Cyanidin-3-O-glucoside showed the highest relative area. It has been reported as one of the most common naturally occurring anthocyanins. It has been found in black grains [9], purple potatoes [41], and several berries [42]. It exhibits significant antioxidant, anti-inflammatory, and antidiabetic activity. Moreover, it has cytoprotective effects against several oxidative stress-induced disorders [43].

Conversely, some authors identified petunidin-3-O-glucoside, delphinidin-3-O-glucoside, and malvidin-3-O-glucoside in common and black bean ethanolic extracts [29,44]. 

## 4. Conclusions

The application of the RSM enabled the development of a UAE procedure for the purposes of maximizing the content of phenolic compounds in aqueous ethanol extracts from black beans. Among the selected variables, solvent concentration and extraction time significantly affected TAC, while time and solvent/sample ratio greatly influenced TPC. Among the compounds identified in the extracts, pigmented phenolics (such as cyanidin-3-O-glucoside and peonidin-3-O-glucoside) and phenolic acids (such as caffeic, sinapic, and t-ferulic acids) were present.

The present study lays the groundwork for a reliable estimation of phenolic content in black bean by using green solvents such as ethanol and green technologies such as ultrasounds. In the context of understanding the contribution of this ecotype to the dietary intake of phenolic compounds, further investigations are necessary to consider the possible effect of black bean processing on phytochemical content.

## Figures and Tables

**Table 1:** Experimental design (Box–Behnken) applied to optimize the extraction of phenolic compounds from black beans. Real values adopted for each factor in the UAE of phenolic compounds and coded values (in parentheses) are shown.

Run	Factors		
	Solvent Concentration (%)	Time (min)	SSR (mL g^−1^)
1	50 (−1)	20 (0)	20 (−1)
2	65 (0)	20 (0)	35 (0)
3	50 (−1)	30 (+1)	35 (0)
4	50 (−1)	20 (0)	50 (+1)
5	65 (0)	20 (0)	35 (0)
6	50 (−1)	10 (−1)	35 (0)
7	80 (+1)	20 (0)	50 (+1)
8	65 (0)	30 (+1)	20 (−1)
9	65 (0)	20 (0)	35 (0)
10	65 (0)	10 (−1)	50 (+1)
11	80 (+1)	10 (−1)	35 (0)
12	65 (0)	10 (−1)	20 (−1)
13	80 (+1)	20 (0)	20 (−1)
14	65 (0)	20 (0)	35 (0)
15	65 (0)	30 (+1)	50 (+1)
16	80 (+1)	30 (+1)	35 (0)
17	65 (0)	20 (0)	35 (0)

SSR: solvent/sample ratio.

**Table 2:** Total Anthocyanin Content (TAC) and Total Phenolic Content (TPC) of the extracts.

Run	TAC (mg C3GE 100 g^−1^ dm)	TPC (mg GAE 100 g^−1^ dm)
1	40.76 ± 0.51	31.95 ± 1.35
2	49.67 ± 0.49	42.87 ± 0.84
3	62.93 ± 0.65	43.11 ± 0.74
4	40.67 ± 0.24	39.48 ± 0.91
5	53.89 ± 0.44	41.30 ± 0.55
6	55.92 ± 0.77	39.92 ± 0.22
7	25.41 ± 0.23	36.29 ± 0.15
8	86.20 ± 0.63	37.95 ± 0.09
9	60.62 ± 0.79	40.32 ± 0.38
10	73.05 ± 0.61	46.40 ± 1.19
11	32.77 ± 0.30	33.88 ± 0.53
12	57.81 ± 0.48	40.34 ± 0.24
13	39.51 ± 0.39	34.09 ± 0.85
14	49.95 ± 0.43	44.74 ± 0.21
15	72.58 ± 0.56	57.87 ± 2.19
16	63.99 ± 0.22	41.39 ± 0.26
17	60.59 ± 0.44	45.83 ± 0.98

Values are expressed as mean (*n* = 3) on a dry matter basis.

**Table 3:** Regression analysis of the second-order polynomial models for optimizing the two responses (TAC and TPC).

Factor	Coefficient (β)	
	TAC	TPC
Intercept	54.94 **	43.01 **
Linear		
X_1_	−4.83 *	−1.10
X_2_	8.27 *	2.47 *
X_3_	−1.57	4.46 *
Quadratic		
$X_{1}^{2}$	−18.43 **	−6.81 *
$X_{2}^{2}$	17.39 **	3.37 *
$X_{3}^{2}$	0.08	−0.75
Interaction		
X_1_ X_2_	6.05 *	1.08
X_1_ X_3_	−3.51	−1.34
X_2_ X_3_	−7.21 *	3.46 *
R^2^	0.9572	0.8956
Adj. R^2^	0.9023	0.7613
F-value (model)	17.41 **	6.67 *
F-value (lack of fit)	0.55	0.20

X_1_: solvent concentration; X_2_: extraction time; X_3_: solvent/sample ratio; R^2^: coefficient of determination; Adj. R^2^: adjusted R^2^. Level of significance: * *p* < 0.05; ** *p* < 0.001.

## Data Availability

The data presented in this study are available upon request from the corresponding author.
